# Association between systemic immunity-inflammation index and hypertension in US adults from NHANES 1999–2018

**DOI:** 10.1038/s41598-024-56387-6

**Published:** 2024-03-07

**Authors:** Ying Chen, Yanping Li, Mengqiong Liu, Wenxing Xu, Shan Tong, Kai Liu

**Affiliations:** 1 Medical Laboratory Center, Hainan General Hospital (Hainan Affiliated Hospital of Hainan Medical University), Haikou, 570311 Hainan China; 2 Hainan General Hospital (Hainan Affiliated Hospital of Hainan Medical University), Haikou, 570311 Hainan China; 3 Geriatric Center, Hainan General Hospital (Hainan Affiliated Hospital of Hainan Medical University), Haikou, 570311 Hainan China

**Keywords:** Systemic immunity-inflammation index, High blood pressure, NHANES, Risk factors, Weighted logistic regression, Predictive markers, Hypertension

## Abstract

Hypertension is a disease closely related to inflammation, and the systemic immunity-inflammation index (SII) is a new and easily detectable inflammatory marker. We aimed to investigate the association between SII and hypertension risk in a adult population in the US. We utilized data from the National Health and Nutrition Examination Survey spanning from 1999 to 2018, incorporating comprehensive information from adults reporting hypertension. This included details on blood pressure monitoring, complete blood cell counts, and standard biochemical results. The SII was computed as the platelet count multiplied by the neutrophil count divided by the lymphocyte count. We employed a weighted multivariate logistic regression model to examine the correlation between SII and hypertension. Subgroup analyses were conducted to explore potential influencing factors. Furthermore, smooth curve fitting and two-piecewise logistic regression analysis were employed to describe non-linear relationships and identify inflection points. This population-based study involved 44,070 adults aged 20–85 years. Following Ln-transformation of the SII, multivariable logistic regression revealed that, in a fully adjusted model, participants in the highest quartile of Ln(SII) had a 12% increased risk of hypertension compared to those in the lowest quartile, which was statistically significant (OR:1.12; 95% CI 1.01, 1.24; *P* < 0.001), with a* P* for trend = 0.019. Subgroup analysis indicated no significant interactions between Ln(SII) and specific subgroups except for the body mass index subgroup (all *P* for interaction > 0.05). Additionally, the association between Ln(SII) and hypertension displayed a U-shaped curve, with an inflection point at 5.89 (1000 cells/μl). Based on this research result, we found a U-shaped correlation between elevated SII levels and hypertension risk in American adults, with a inflection point of 5.89 (1000 cells)/μl). To validate these findings, larger scale prospective surveys are needed to support the results of this study and investigate potential mechanisms.

High blood pressure affects a significant portion of the global population and remains a crucial public health issue due to its association with cardiovascular morbidity and mortality^1–3^. While traditional risk factors for hypertension have been established, emerging evidence suggests that inflammation plays a pivotal role in the onset and progression of this condition^4^. Understanding the dynamic interplay between inflammation and hypertension is paramount for identifying novel biomarkers that can enhance risk stratification and provide insights for targeted therapeutic interventions.

The systemic immunity-inflammation index (SII) is a novel comprehensive inflammation biomarker based on lymphocyte, neutrophil, and platelet counts, which has been extensively studied since its inception^5–7^. Numerous studies have highlighted the close association of SII with conditions such as hyperlipidemia^8^, heart failure^9^, and mortality due to cardiogenic shock^10^. It is widely believed that the onset and maintenance of elevated blood pressure are closely related to low-grade inflammation^11^. Furthermore, despite effective blood pressure management, patients with hypertension still face cardiovascular risks. This underscores the persistence of residual cardiovascular risk, which may be associated with immune cell activation and chronic inflammation^12^.

Currently, there is research on the relationship between SII and hypertension, but the sample sizes are small, and the research conclusions vary^13–15^. Therefore, there is an urgent need to investigate the precise relationship between SII and the risk of developing hypertension in a representative large sample population. The National Health and Nutrition Examination Survey (NHANES) is a program designed to assess the health and nutritional status of adults and children in the United States. The findings from this survey are utilized to determine the prevalence of major diseases and risk factors for diseases. Consequently, we conducted a cross-sectional study using a large sample from NHANES, focusing on a population aged 20 to 85 years. The aim is to explore the relationship between SII and the prevalence of hypertension in different population cohorts in the United States. In addition, we hypothesize a positive correlation between SII and the risk of hypertension in American adults, and further validate this through these studies.

## Methods

### Study population

**Figure 1:**
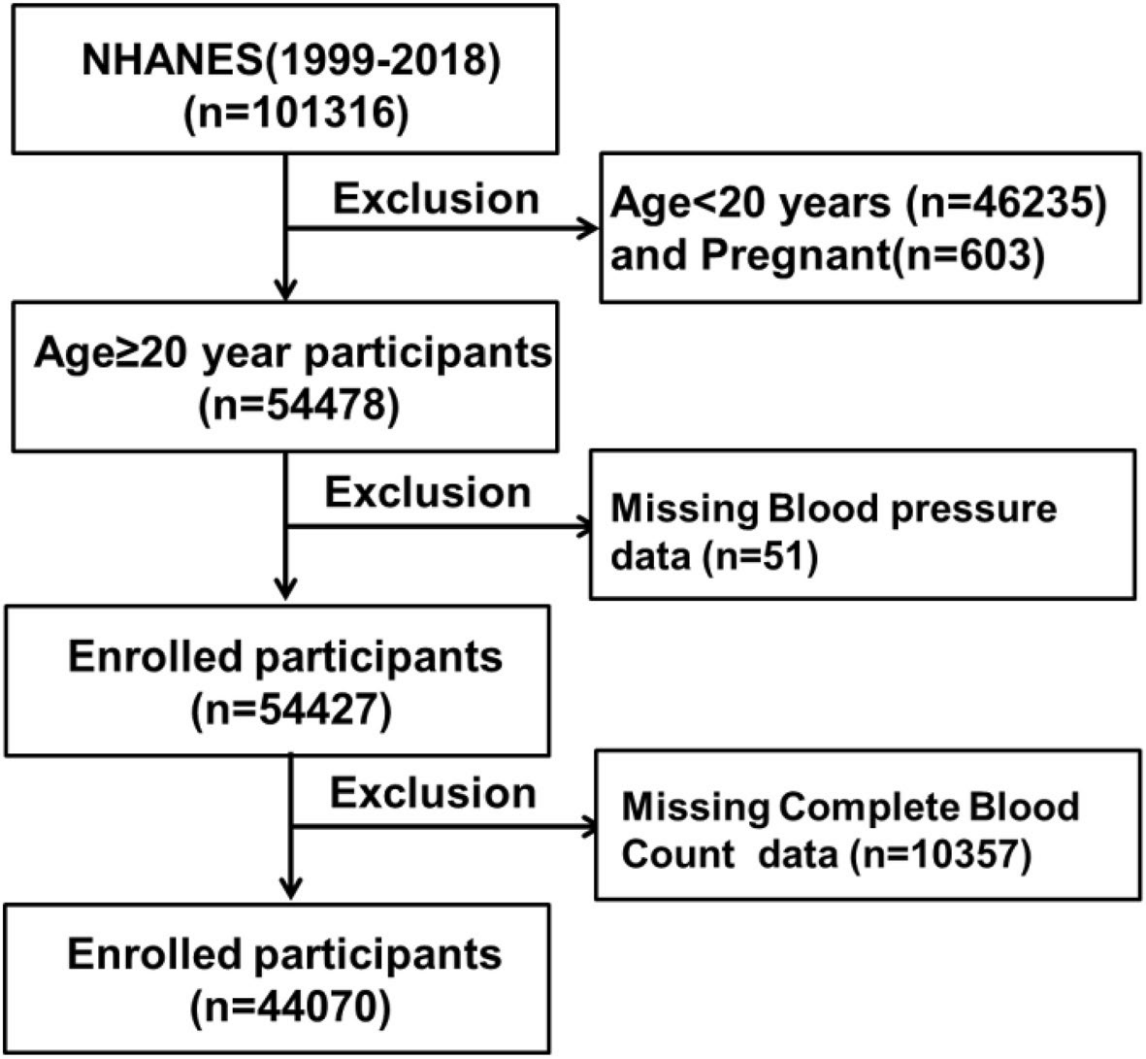
Flowchart of participant selection. NHANES, National Health and Nutrition Examination Survey.

To ensure the representativeness of the sample, participants in the NHANES survey were selected using a stratified multi-stage probability sampling method. In our study, data from ten consecutive cycles of NHANES conducted between 1999 to 2018 were utilized. Participants included in this study had complete demographic data, standard physical measurements, biochemical indicators, and Medical Conditions information. Exclusion criteria were as follows: (1) age < 20 years, (2) pregnancy, (3) missing key clinical records, including failure to participate in the blood pressure survey questionnaire and absence of any blood pressure measurement data, and missing Complete Blood Count with 5-part Differential data. This study adhered to the STROBE Checklist for observational studies (https://www.strobe-statement.org/checklists/). The recruitment process for all enrolled participants is shown in Fig. 1.

### Definition of hypertension

Perform three or even four blood pressure measurements (systolic and diastolic blood pressure) during mobile examination centers (MEC) and home examinations of all eligible individuals using mercury sphygmomanometers. Participants aged 50 and above or under one year who are unable to travel to MEC will undergo a brief exam at home. Blood pressure measurement is performed by a MEC examiner. The technique used to obtain BP follows the latest recommendations of the American Heart Association Human Blood Pressure Determination by sphygmomanometers^16^. The blood pressure measurements of participants from 2017 to 2020 were conducted using the digital upper arm electronic measurement device Omron HEM-907XL, with three consecutive blood pressure measurements taken at 60 s intervals. This device has previously been validated by the Association for the Advancement of Medical Instruments (AAMI) and the European Society for Hypertension international protocol for measuring blood pressure in individuals aged 13 years and above. The average value of all available measurements was recorded. According to the definition of the European Society of Cardiology^17^, participants were defined as having hypertension with (I) average systolic blood pressure ≥ 140 mmHg, (II) average diastolic blood pressure ≥ 90 mmHg, (III) current use of anti-hypertensive medications, (IV) participants with a self-reported hypertension^18,19^.

### Definition of systemic immunity-inflammation index (SII)

According to NHANES, using an automated hematological analysis device (Coulter DxH 800 analyzer), lymphocyte, neutrophil, and platelet counts were measured and reported as 1000 cells/mL. SII is derived from blood samples, which undergo rigorous laboratory testing in accordance with standardized sampling protocols to ensure the validity and comparability of the data. Blood samples are typically collected on survey vehicles or at specified sampling sites, followed by processing and testing within the laboratory. SII was calculated based on the results of the complete blood count test. Platelet count, neutrophil count, and lymphocyte count were measured in 1,000 cells/μl. The calculation of SII is based on a precise formula: SII = platelet count × neutrophil count/lymphocyte count^20–22^.

### Covariates

Our study also selected possible factors that may affect the association between clinical relevance based SII and hypertension, including age (year), gender (male/female), race (Mexican American/Other Hispanic/Non-Hispanic White/Non-Hispanic Black/Other Race), body mass index (BMI, kg/m^2^), Waist circumference, education level, family poverty income ratio (PIR), smoking, alcohol use, chronic congestive heart failure, coronary heart disease, stroke, weak/failing kidneys, diabetes, angina pectoris, heart attack, chronic bronchitis, emphysema, thyroid disease, glucose, total cholesterol, triglycerides, uric acid, low-density lipoprotein cholesterol, and high-density lipoprotein cholesterol, alanine aminotransferase, aspartate aminotransferase, blood urea nitrogen, calcium, cholesterol, creatinine, glucose, triglycerides, uric acid, sodium ,potassium,globulin,albumin^8,9,17–19^.

### Statistical analysis

Weights are created in NHANES to account for the complex survey design (including oversampling), survey non-response, and post-stratification adjustment to match total population counts from the Census Bureau. The calculation method for weights can be found in Supplementary Document 1. Continuous variables were presented as the mean ± standard deviation (SD) (normal distribution), or the median (interquartile range) (skewed distribution). We used Kolmogorov–Smirnov test to assess the normality. Categorical variables were presented as the number (percentage). Differences in baseline variables were tested using weighted t-test, weighted chi-square test, or Fisher's exact test. For skewed distributed continuous variables, between-group comparisons are conducted using the weighted Wilcoxon rank-sum test.

A weighted logistic regression model was used to investigate the relationship between SII and hypertension. Model 1 was not adjusted for any covariates. Model 2 was adjusted for age, gender. Model 3 was adjusted for age, gender, race, family poverty income ratio, education level, alcohol use, smoking, body mass index(BMI), waist circumference, asthma, arthritis, congestive heart failure, coronary heart disease, stroke, weak/failing kidneys, diabetes, angina pectoris, heart attack, chronic bronchitis, emphysema, thyroid disease, liver disease, cancer, stroke, albumin, glucose, total cholesterol, triglycerides, uric acid, low-density lipoprotein cholesterol, and high-density lipoprotein cholesterol, alanine aminotransferase, aspartate aminotransferase, blood urea nitrogen, calcium, cholesterol, creatinine, glucose, triglycerides, uric acid, sodium, potassium, globulin.

Restricted cubic spline analysis (RCS) (with three knots) was used to evaluate the nonlinear associations between SII and the risk of hypertension, the median value of SII was used as a reference. Two-piecewise Logistic regression analysis model was used to examine the relationship between SII and hypertension and the inflection point. Finally, we used subgroup analysis to divide the participants into different levels, including age, gender, race, education level, body mass index, waist, diabetes, coronary heart disease, diabetes, stroke, smoking, alcohol use, weak/failing kidneys added interaction terms to test for heterogeneity among subgroups. All statistical analyses were performed in R software, version 4.3.1 and P < 0.05 was regarded as significant.

### Ethics approval and consent to participate

The new ethic statement as follows: The NCHS Ethics Review Board protects the rights and welfare of NHANES participants. The NHANES protocol complies with the U.S. Department of Health and Human Services Policy for the Protection of Human Research Subjects. NCHS IRB/ERB Protocol Number or Description (https://www.cdc.gov/nchs/nhanes/irba98.htm). Ethical review and approval were waived for this study as it solely used publicly available data for research and publication. Informed consent was obtained from all subjects involved in the NHANES. This study was deemed exempt from review by the Ethics Committee of Hainan General Hospital.

## Results

### Characteristics of the study population

A total of 44,070 adult participants from NHANES (1999–2018) were included in this study. Among them, 15,234 (34.6%) had hypertension. Participants were grouped according to the quartile of Ln (SII), and the crude prevalence of hypertension in the first, second, third, and fourth quartiles were 3722 (33.8%), 3610 (32.8%), 3788 (34.4%), and 4114 (37.3%), respectively (Supplementary Fig. 1). Significant differences were observed between the hypertension (HTN) and normal blood pressure (NON-HTN) groups in terms of demographic and comorbidity factors (Table 1). With the exception of serum cholesterol, sodium, and HDL-cholesterol, all other biochemical markers showed significant statistical differences between the two groups (Table 1). The SII in the hypertension group was significantly higher than that in the normal blood pressure group (Table 1).

**Table 1:** Weighted comparison in basic characteristics.

Variables	Overall	HTN	NON-HTN	*P*
	n = 44,070	n = 15,234	n = 28,836	
Age (year)	49.79 (18.18)	60.33 (15.26)	44.22 (17.09)	< 0.001
Gender (%)				0.033
Male	21,456 (48.69)	7253 (47.61)	14,203 (49.25)	
Female	22,614 (51.31)	7981 (52.39)	14,633 (50.75)	
Race/Ethnicity (%)				< 0.001
Mexican American	7950 (18.04)	2123 (13.94)	5827 (20.21)	
Other Hispanic	3584 (8.13)	1135 (7.45)	2449 (8.49)	
Non-Hispanic White	20,094 (45.60)	7169 (47.06)	12,925 (44.82)	
Non-Hispanic Black	8879 (20.15)	3852 (25.29)	5027 (17.43)	
Other race	3563 (8.08)	955 (6.27)	2608 (9.04)	
Education level (%)				< 0.001
Less than 11th grade	12,308 (27.93)	4767 (31.29)	7541 (26.15)	
High school or equivalent	10,148 (23.03)	3707 (24.33)	6441 (22.34)	
College or AA degree	12,279 (27.86)	4139 (27.17)	8140 (28.23)	
College or above	9335 (21.18)	2621 (17.20)	6714 (23.28)	
Smoking (%)				< 0.001
Yes	20,352 (46.18)	7690 (50.48)	12,662 (43.91)	
No	23,718 (53.82)	7544 (49.52)	16,174 (56.09)	
Alcohol use (%)				< 0.001
Yes	28,468 (64.60)	9402 (61.72)	19,066 (66.12)	
No	15,602 (35.40)	5832 (38.28)	9770 (33.88)	
Antihypertensive drugs (%)				< 0.001
Yes	13,032 (29.57)	13,032 (85.55)	0 (0.00)	
No	31,038 (70.43)	2202 (14.45)	28,836 (100.00)	
Diabetes (%)				< 0.001
Yes	5411 (12.28)	3701 (24.29)	1710 (5.93)	
No	36,436 (82.68)	10,300 (67.61)	26,136 (90.64)	
Boderline	2223 (5.04)	1233 (8.09)	990 (3.43)	
Asthma (%)				< 0.001
Yes	5792 (13.14)	2340 (15.36)	3452 (11.97)	
No	38,278 (86.86)	12,894 (84.64)	25,384 (88.03)	
Arthritis (%)				< 0.001
Yes	11,713 (26.58)	6716 (44.09)	4997 (17.33)	
No	32,357 (73.42)	8518 (55.91)	23,839 (82.67)	
Congestive heart failure (%)				< 0.001
Yes	1446 (3.28)	1117 (7.33)	329 (1.14)	
No	42,624 (96.72)	14,117 (92.67)	28,507 (98.86)	
Cardiovascular disease (%)				< 0.001
Yes	1841 (4.18)	1343 (8.82)	498 (1.73)	
No	42,229 (95.82)	13,891 (91.18)	28,338 (98.27)	
Angina pectoris (%)				< 0.001
Yes	1293 (2.93)	947 (6.22)	346 (1.20)	
No	42,777 (97.07)	14,287 (93.78)	28,490 (98.80)	
Heart attack (%)				< 0.001
Yes	1939 (4.40)	1376 (9.03)	563 (1.95)	
No	42,131 (95.60)	13,858 (90.97)	28,273 (98.05)	
Stroke (%)				< 0.001
Yes	1646 (3.73)	1225 (8.04)	421 (1.46)	
No	42,424 (96.27)	14,009 (91.96)	28,415 (98.54)	
Emphysema (%)				< 0.001
Yes	910 (2.06)	537 (3.53)	373 (1.29)	
No	43,160 (97.94)	14,697 (96.47)	28,463 (98.71)	
Thyroid disease (%)				< 0.001
Yes	613 (1.39)	280 (1.84)	333 (1.15)	
No	43,457 (98.61)	14,954 (98.16)	28,503 (98.85)	
Chronic bronchitis (%)				< 0.001
Yes	2548 (5.78)	1289 (8.46)	1259 (4.37)	
No	41,522 (94.22)	13,945 (91.54)	27,577 (95.63)	
Liver disease (%)				< 0.001
Yes	1609 (3.65)	814 (5.34)	795 (2.76)	
No	42,461 (96.35)	14,420 (94.66)	28,041 (97.24)	
Cancer (%)				< 0.001
Yes	3988 (9.05)	2233 (14.66)	1755 (6.09)	
No	40,082 (90.95)	13,001 (85.34)	27,081 (93.91)	
Weak/failing kidneys (%)				< 0.001
Yes	1219 (2.77)	866 (5.68)	353 (1.22)	
No	42,851 (97.23)	14,368 (94.32)	28,483 (98.78)	
Family poverty income ratio,%	2.54 (1.62)	2.47 (1.59)	2.58 (1.64)	< 0.001
Systolic pressure, mmHg	123.19 (31.82)	133.18 (34.35)	117.91 (29.05)	< 0.001
Diastolic pressure, mmHg	70.25 (19.09)	71.35 (20.77)	69.66 (18.11)	< 0.001
Body mass index, kg/m2	28.86 (6.71)	30.83 (7.21)	27.82 (6.18)	< 0.001
Waist Circumference (cm)	98.57 (15.89)	104.25 (15.76)	95.56 (15.11)	< 0.001
Lymphocyte count (1000 cells/μl)	2.15 (1.26)	2.13 (1.44)	2.16 (1.16)	0.080
Neutrophil count (1000 cells/μl)	4.30 (1.78)	4.38 (1.90)	4.26 (1.71)	< 0.001
Platelet count (1000 cells/uL)	253.04 (67.95)	249.79 (71.53)	254.76 (65.92)	0.019
SII (1000 cells/μl)	344.00 [484.00,683.69]	506.79 [364.76,714.00]	479.45 [349.53,668.11]	< 0.001
Ln (SII)	6.18 (0.55)	6.20 (0.57)	6.17 (0.53)	< 0.001
Albumin (g/L)	42.54 (3.56)	41.93 (3.44)	42.86 (3.59)	< 0.001
ALT (U/L)	21 [16, 28]	22 [17, 30]	21 [16, 28]	< 0.001
AST (U/L)	23 [19, 28]	24 [20, 29]	23 [19, 27]	< 0.001
Blood urea nitrogen (mmol/L)	4.87 (2.18)	5.60 (2.76)	4.48 (1.67)	< 0.001
Total calcium (mmol/L)	2.36 (0.09)	2.36 (0.10)	2.35 (0.09)	< 0.001
Serum cholesterol (mmol/L)	5.07 (1.08)	5.05 (1.12)	5.08 (1.06)	0.975
Creatinine(umol/L)	74.26 [61.88, 88.40]	79.56 [67.18, 93.70]	73.37 [61.90,87.52]	< 0.001
Glucose (mmol/L)	5.65 (2.15)	6.18 (2.60)	5.37 (1.81)	< 0.001
Triglycerides (mmol/L)	1.36 [0.90,2.08]	1.57 [1.05,2.36]	1.25 [0.84,1.94]	< 0.001
Uric acid (umol/L)	323.18 (86.23)	347.41 (91.25)	310.38 (80.57)	< 0.001
Sodium (mmol/L)	139.14 (2.38)	139.16 (2.61)	139.13 (2.25)	0.244
Potassium (mmol/L)	4.00 (0.35)	4.01 (0.40)	3.99 (0.32)	< 0.001
Globulin (g/L)	29.63 (4.69)	30.12 (4.99)	29.37 (4.50)	< 0.001
LDL- cholesterol (mg/dL)	115.48 (35.61)	114.10 (35.67)	116.21 (35.56)	< 0.001
HDL- cholesterol (mg/dL)	52.74 (15.99)	52.72 (15.87)	52.75 (16.05)	0.820

Mean ± SD for continuous variables: P value was calculated by weighted t test. % for categorical variables: P value was calculated by weighted chi-square test. Median [interquartile range] for continuous variables: P value was calculated by Wilcoxon rank-sum test.

*ALT* Alanine Aminotransferase, *AST* Aspartate Aminotransferase. *LDL- cholesterol* low-Density Lipoprotein Cholesterol, *HDL- cholesterol* high-Density Lipoprotein Cholesterol, *SII* systemic immunity-inflammation index, *HTN* hypertension.

### Association between SII and hypertension

**Figure 2:**
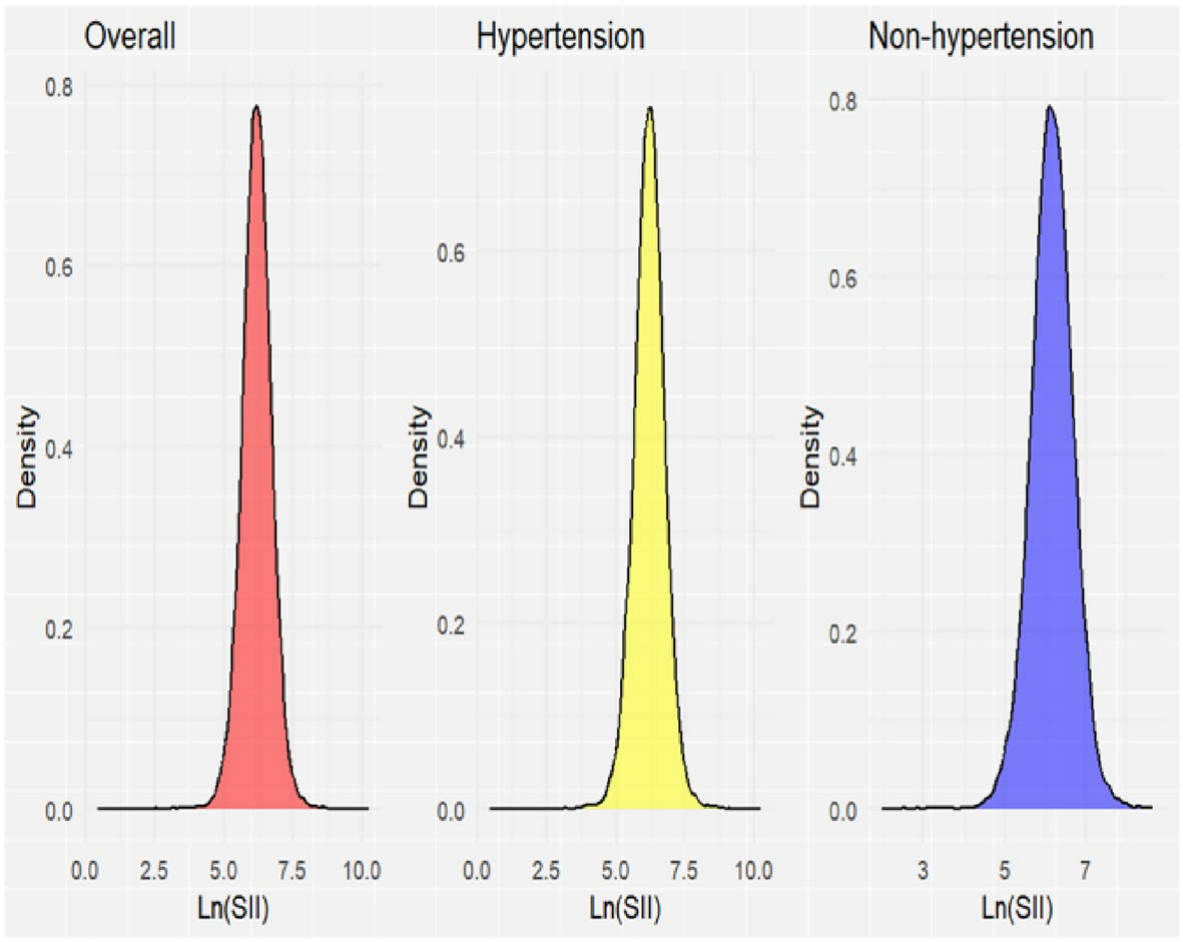
The overall distribution of Ln(SII) and distribution of Ln(SII) in individuals with hypertension and without hypertension.

Referring to the methods of multiple recent studies^23–27^, in order to correct the skewed distribution of SII to a normal distribution and amplify the association effect between SII and hypertension risk, we performed Ln logarithmic transformation on SII (Supplementary Fig. 2). The distribution of Ln (SII) after transformation is shown in Fig. 2. Our study results indicate a significant association between higher SII and increased likelihood of hypertension (Table 2). Both Model 1 (OR: 1.21; 95% CI 1.14–1.28; *P* < 0.001) and Model 2 demonstrated this correlation to be significant (OR: 1.17; 95% CI 1.11–1.25; *P* < 0.001). In Model 3, a significant association between SII and hypertension persisted (OR: 1.10; 95% CI 1.03–1.17; *P* < 0.001), indicating a 10% increase in the risk of hypertension for each unit increase in Ln(SII) levels. When using Ln(SII) as a categorical variable for sensitivity analysis, compared to subjects with the lowest tetile of Ln(SII), those with the highest SII had a statistically significant 12% increase in the risk of hypertension (OR: 1.12; 95% CI 1.01–1.24; *P* = 0.027) (Table 2). The results of unweighted crude logistic regression analysis also have similar correlations, as shown in Supplementary Table 1.

**Table 2:** Weighted Logistic Regression Analysis of Ln (SII) and Hypertension.

	Model 1		Model 2		Model 3	
	OR(95%CI)	P	OR(95%CI)	P	OR(95%CI)	P
Continuous	1.21 (1.14, 1.28)	< 0.001	1.17 (1.11, 1.25)	< 0.001	1.10 (1.03, 1.17)	< 0.001
Categories						
Tertile 1	Ref		Ref		Ref	
Tertile 2	0.99 (0.92, 1.07)	0.850	0.99 (0.91, 1.08)	0.878	1.03 (0.94, 1.13)	0.525
Tertile 3	1.10 (1.01, 1.19)	0.026	1.11 (1.01, 1.21)	0.029	1.09 (0.98, 1.20)	0.101
Tertile 4	1.29 (1.19, 1.41)	< 0.001	1.23 (1.12, 1.35)	< 0.001	1.12 (1.01, 1.24)	0.027
*P* for trend		< 0.001		< 0.001		0.019

*SII* systemic immunity-inflammation index.

Ln (SII) Tertile 1: 0–5.840; Tertile 2:5.841–6.181; Tertile 3:6.182–6.527; Tertile 4: > 6.527.

### Smooth curve fitting, threshold effect analyses between SII and hypertension

**Figure 3:**
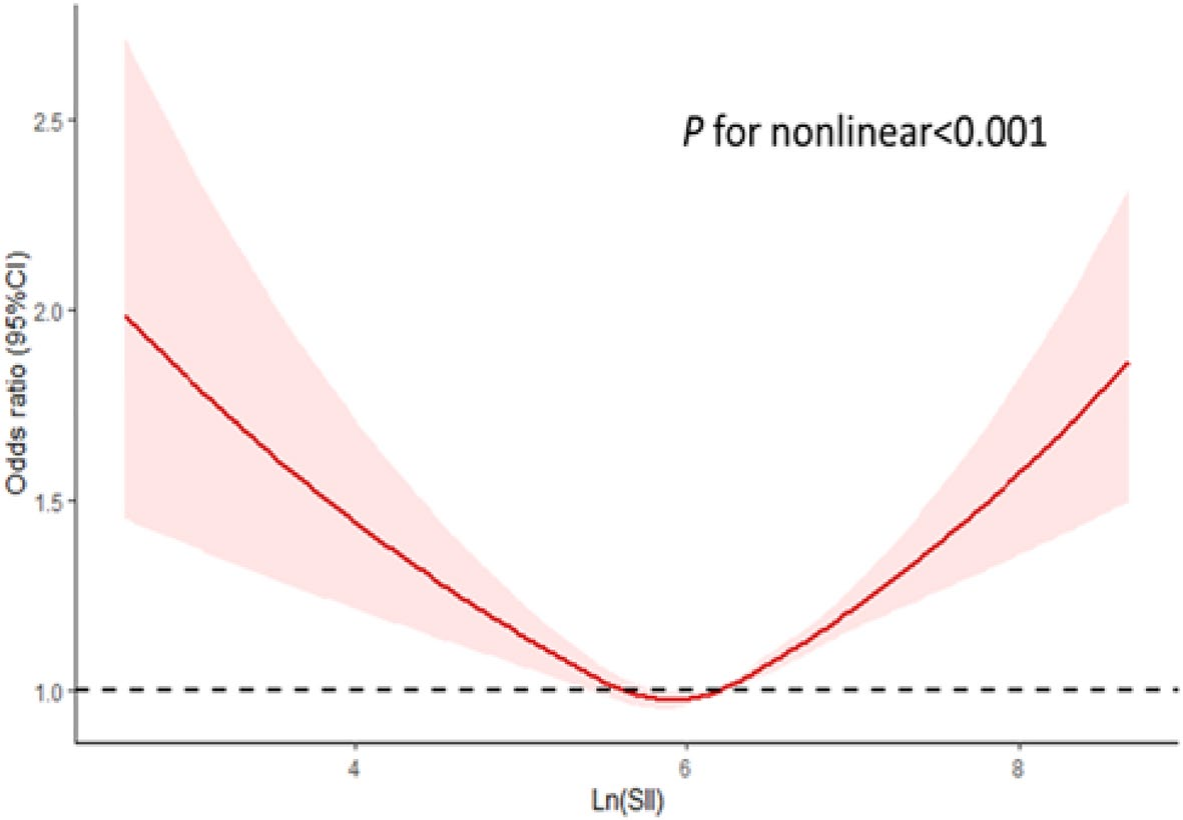
Restricted cubic spline analysis of the relationship between systemic immunity-inflammation index and hypertension.

To further explore the relationship between SII and hypertension, we conducted a threshold effect analysis using smooth curve fitting (Fig. 3). We identified a U shaped relationship between SII and the risk of hypertension, with a inflection point at 5.89 (1000 cells/μl). When Ln(SII) levels were below 5.89 (1000 cells/μl), the prevalence of hypertension decreased with increasing Ln(SII) (OR: 0.95; 95% CI 0.92–0.97; *P* < 0.001).However, when Ln(SII) exceeded 5.89 (1000 cells/μl), the risk of hypertension increased with the elevation of Ln(SII) (OR: 1.12; 95% CI 1.09–1.15; *P* < 0.001) (Table 3).

**Table 3:** Analysis of the threshold effect of SII on hypertension by two-piece linear regression model.

Inflection point	OR (95% CI)	P
≤ 5.89	0.95 (0.92, 0.97)	< 0.001
> 5.89	1.12 (1.09, 1.15)	< 0.001
Log likelihood ratio tests		< 0.001

### Subgroup analyses

**Figure 4:**
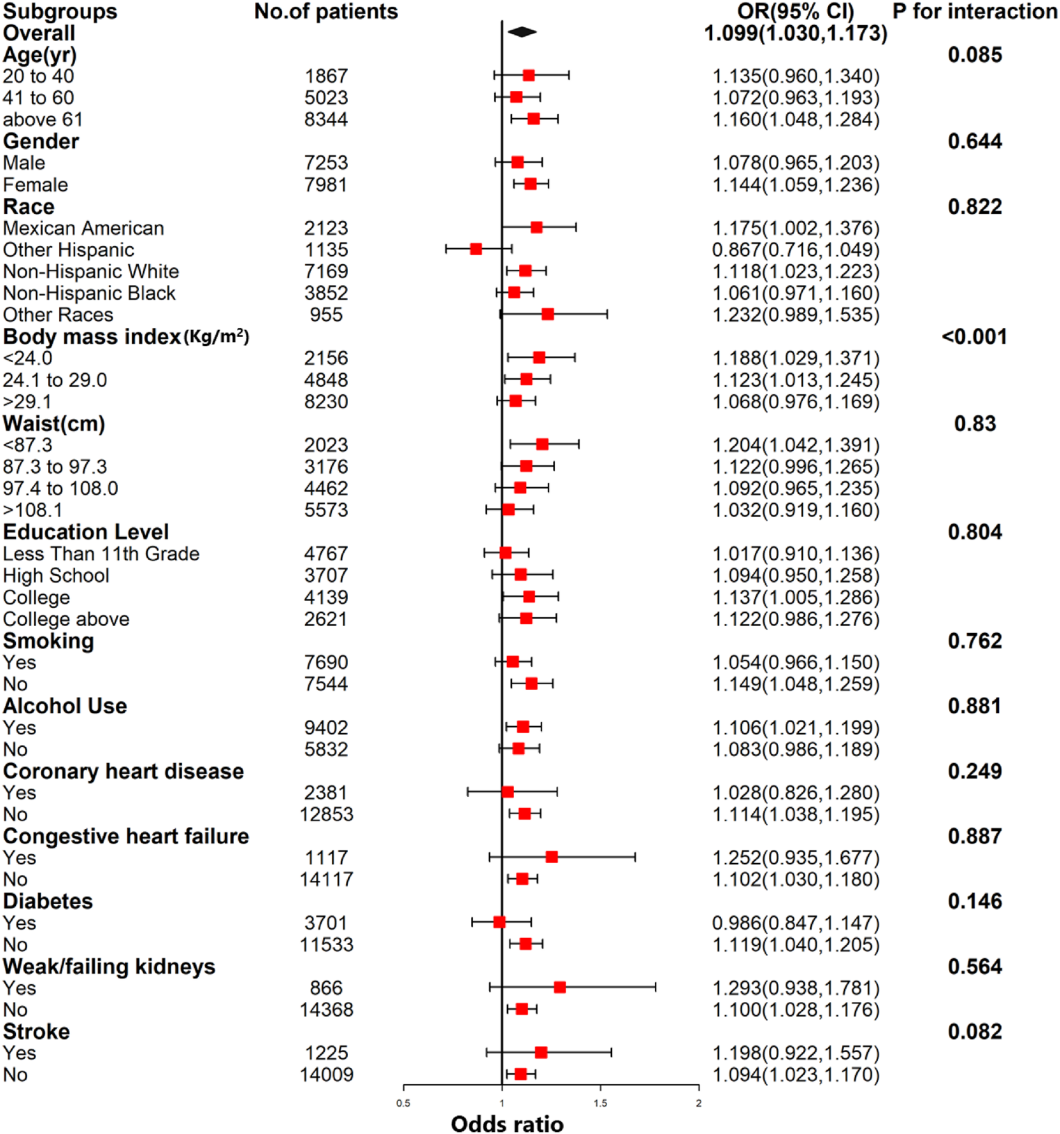
Subgroup analysis for the association between systemic immunity-inflammation index and hypertension.

To assess the stability of the association between SII and hypertension across various subgroups, we conducted a subgroup analysis. Interaction tests revealed no statistically significant differences in the association between SII and hypertension across subgroups (Fig. 4). This indicates that factors such as gender (male/female), race (Mexican American/Other Hispanic/Non-Hispanic White/Non-Hispanic Black/Other race), age (20–40/41–60/ ≥ 61 years), education level (Less Than 11th Grade/High School or Equivalent/College or AA degree/College or above), smoking (yes/no), alcohol use (yes/no), diabetes (yes/no), coronary heart disease (yes/no), congestive heart failure (yes/no), stroke (yes/no), and weak/failing kidneys (yes/no) did not significantly affect this positive association (all *P* for all interaction > 0.05). However, in the BMI (< 24/24.1–29/ > 29.1 kg/m^2^) subgroup, there was a significant intergroup interaction (P for interaction < 0.001).Furthermore, a weighted logistic regression subgroup analysis based on the quartiles of waist circumference showed no interaction between the groups (P for interaction = 0.082). This suggests that the association between SII and hypertension remained consistent across subgroups, indicating high stability and reliability.

## Discussion

In this comprehensive survey involving 44,070 adult participants in the United States, we discovered a noteworthy correlation between the systemic immunity-inflammation index (SII) levels and the prevalence of hypertension. Our exploration of these findings, employing in-depth subgroup analysis and interaction tests, revealed a similar trend in this association. The relationship between SII and hypertension exhibits a U-shaped pattern. When Ln(SII) is below 5.89 (1000 cells/μl), it is associated with a decreased risk of hypertension; however, once this threshold is surpassed, Ln(SII) becomes associated with an increased risk of hypertension. These findings validate and deepen the hypothesis initially proposed in this study, highlighting the complex interaction between SII and cardiovascular health, thus providing important insights to advance our understanding of the immune-inflammatory processes in the field of cardiovascular-related diseases.

Based on existing literature, our study represents the largest sample size investigation to date on the relationship between SII and hypertension. In a recent study by Akyüz et al.^14^, the SII levels in the non-dipper type hypertension group were higher than those in the dipper type hypertension group. SII was identified as an independent predictive factor for non-dipper type hypertension, and elevated SII values in hypertensive patients could serve as an early warning parameter for identifying non-dipper type hypertension. In a retrospective study involving 153 cases, it was found that newly diagnosed, untreated hypertensive women exhibited higher SII levels than men. Additionally, SII in this population was found to be age-related^13^. Another study by Saylik et al. demonstrated that SII is elevated in patients with morning blood pressure elevation and is closely associated with morning blood pressure elevation^28^. Karakayali's research^15^ suggests that the SII level in the reverse-dipper type hypertension group is higher than that in the dipper and non-dipper type hypertension groups. Furthermore, SII serves as an independent predictive indicator for newly diagnosed patients with reverse-dipper hypertension. While Akyüz^14^ and Karakayali's^15^ studies revealed subtle differences in research results, indicating potential controversy regarding the association between SII and hypertension types, our study aligns with the consensus that there is an association between SII and hypertension. Although NHANES data do not allow for the subtyping of hypertensive patients, the results from these studies at least suggest a general correlation between SII and hypertension. Discrepancies in research results regarding the association between SII and hypertension subtypes may be attributed to factors such as sample size, ethnic differences, and further research with larger, representative populations is warranted to confirm these findings.

In studies involving participants from the National Health and Nutrition Examination Survey (NHANES), a close association between the SII and various cardiovascular diseases has been observed. This includes a positive correlation between SII and prevalent aortic calcification in US adults^29^, and higher SII values have been linked to increased incidence of cardiovascular diseases^30^. These findings suggest a potential role for SII in the pathophysiological mechanisms of cardiovascular diseases. Currently, the mechanism underlying the association between SII and hypertension remains incompletely understood. However, numerous research reports have explored the pathological and physiological mechanisms linking inflammation to hypertension. Aboukhter's study extensively discussed how the interaction between inflammation and reactive oxygen species can lead to endothelial damage and dysfunction, ultimately resulting in vascular narrowing and stiffness^31^. This process, combined with abnormal phenotypic changes in vascular smooth muscle cells and increased extracellular matrix deposition, further exacerbates atherosclerosis and vascular non-compliance, ultimately contributing to elevated blood pressure. For over half a century, substantial evidence has implicated inflammation in the pathogenesis of hypertension^32^. Observations have revealed the presence of immune cells within the vascular and renal domains of hypertensive individuals^33^. Several biomarkers associated with inflammation, such as high-sensitivity C-reactive protein^34^, various cytokines^35^, and components of the complement pathways^36^, have been found to be elevated in individuals with hypertension. Emerging insights suggest that hypertension is associated with the activation of inflammatory pathways and alterations in the phenotype of circulating immune cells, particularly within the bone marrow compartment, indicating a potential causal relationship^37^. Araos et al.^38^ demonstrated that neutrophils may contribute to tissue infiltration of immune cells, secrete chemokines/cytokines, and promote pro-inflammatory phenotypes, thereby contributing to the development of arterial hypertension.

A recent study, including 13,742 participants from the National Health and Nutrition Examination Survey (NHANES) spanning from 2017 to 2020, explored the association between the SII and the prevalence of hypertension^24^. The results revealed a J-shaped association between the natural logarithm of SII (LgSII) and hypertension, with the turning point of SII identified at 501.2(Ln-converted to 6.22). On the left side of the turning point, there was no significant correlation between SII and the prevalence of hypertension. However, on the right side (SII ≥ 501.2), the prevalence of hypertension increased with higher SII values (odds ratio [OR]: 3.13; 95% confidence interval [CI] 2.04–4.81). Similar to our research findings, SII is positively correlated with the risk of hypertension when exceeding the inflection point. However, Shi's study concluded that there was no association between SII and the risk of hypertension when SII was below the turning point value. Upon analyzing the methodology of this study, we found that Shi et al. did not conduct weighted analysis when analyzing NHANES data. Given that NHANES employs complex stratified sampling, weighted analysis is crucial in statistical analysis and may influence the results. In our analysis, we also conducted unweighted logistic regression analysis of Ln(SII) and hypertension. Although the overall trend remained non-significant, the trend in the highest quartile of Ln(SII) as a categorical variable showed no statistical difference (*P* for trend = 0.198, Supplementary Table 1).We hypothesized that the possible mechanism behind the U-shaped association may be related to the immune response in hypertensive individuals. When the SII level is below the inflection point value, it may indicate a low-inflammatory state. In this state, protective mechanisms such as maintaining endothelial function^39^, reducing vascular inflammation and endothelial damage^40^, decreasing sympathetic nervous system activity^41^, preserving renal function^42^, thereby reducing the risk of hypertension. However, when the SII surpasses the inflection point, it may signify a high-inflammatory state, which in turn increases the risk of hypertension. A recent study showed that waist circumference is positively correlated with an increase in the incidence of hypertension, and there is a non-linear relationship^17^. Therefore, in our study, waist circumference was adjusted as a covariate and used as a categorical variable for subgroup analysis. The results showed no intergroup interaction in subgroup analysis, and the association between SII and hypertension remained significant. In addition, in the subgroup analysis of our research results, there was a significant intergroup interaction between different BMI subgroups, which may be due to the combined effects of multiple factors such as metabolic health levels, adipose tissue inflammation, lifestyle factors, and gene environment interactions.

While we have conducted a detailed analysis of the impact of SII on hypertension prevalence, there are still some limitations to note in this study. Firstly, this study only utilized a nationally representative sample from the United States. Given significant racial disparities in diet, physical activity, genetic variations, lipid metabolism, and susceptibility to cardiovascular diseases, the generalizability of our conclusions to other populations remains unclear. Secondly, due to the inherent nature of cross-sectional studies, establishing a causal relationship between SII and hypertension is challenging. Further prospective research is needed to determine the exact relationship between different forms of obesity and hypertension. Thirdly, although we adjusted for multiple covariates, we cannot completely rule out the influence of other confounding factors on our results. Additionally, due to limitations in the NHANES database, it is challenging to delineate the risk factors for menopause, which is also a risk factor for hypertension in women^43^. Finally, C-reactive protein as a common inflammatory factor, should be included in the analysis as a covariate. Unfortunately, NHANES has not conducted serum testing for CRP since 2010.

## Conclusions

Our study findings demonstrate a close association between the systemic immunity-inflammation index (SII) levels and the risk of hypertension in US adults, revealing a U-shaped relationship. This suggests that SII may play a complex role in the pathogenesis of hypertension. We also explored the inflection point of SII and differences among different subgroups in terms of hypertension risk. Further exploration of potential mechanisms is needed to better understand the role of SII in the development of hypertension. We recommend larger-scale prospective studies to validate our findings, along with further experimental validation at the cellular and animal levels to elucidate the pathophysiological mechanisms underlying the association between SII and hypertension. These additional studies will contribute to a deeper understanding of the relationship between the immune system and hypertension, ultimately providing more effective strategies for the prevention and treatment of hypertension in the future.

### Supplementary Information

Supplementary Information.

Supplementary Figures.

Supplementary Table 1.

## Data Availability

Publicly available datasets were analyzed in this study. All the raw data used in this study are derived from the public NHANES data portal (https://wwwn.cdc.gov/nchs/nhanes/Default.aspx).
