# Supplementary material for: Association between systemic immunity-inflammation index and hypertension in US adults from NHANES 1999–2018
Source: Sci Rep. 2024 Mar 7;14:5677. doi: 10.1038/s41598-024-56387-6 (PMC10920861; doi:10.1038/s41598-024-56387-6)
Supplement: Supplementary file 1 — Supplementary Information. [file 41598_2024_56387_MOESM1_ESM.pdf]

According to the weight calculation scheme provided by NHANES official(https://wwwn.cdc.gov/nchs/nhanes/tutorials/weighting.aspx).Due to the fact that the Complete Blood Count with 5-Part Differential - Whole Blood and Standard Biochemistry Profile data modules did not provide weight values, we used the Full Sample 2 Year MEC Exam Weight (WTMEC2YR) in the Demographic data module. There are a total of 10 cycles from 1999 to 2018, among which Full Sample 4 Year MEC Exam Weight (WTMEC4YR) was used from 1999 to 2002. The final weight value included in the analysis=(1999-2002 weight value)* 2/10+(2003-2018 weight value) * 1/10.
