# Supplementary material for: Association between systemic immunity-inflammation index and hypertension in US adults from NHANES 1999–2018
Source: Sci Rep. 2024 Mar 7;14:5677. doi: 10.1038/s41598-024-56387-6 (PMC10920861; doi:10.1038/s41598-024-56387-6)
Supplement: Supplementary file 2 — Supplementary Figures. [file 41598_2024_56387_MOESM2_ESM.pdf]

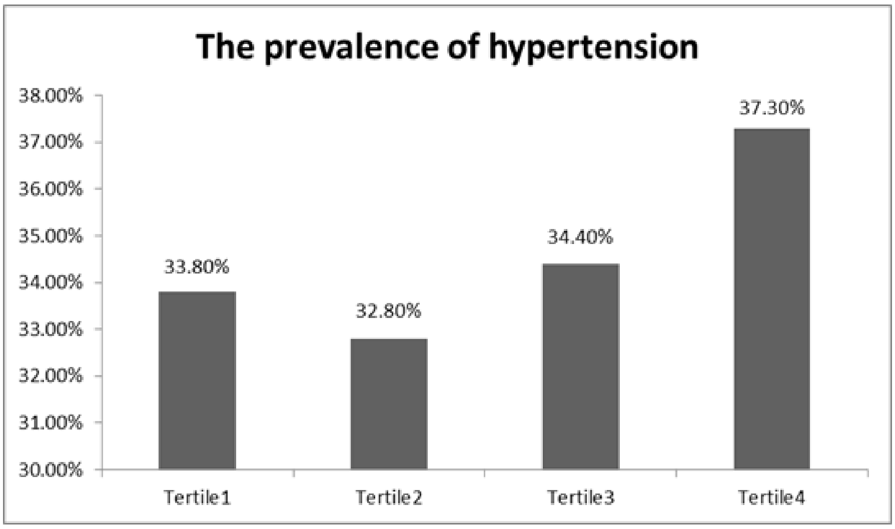

Supplementary Figure 1. The crude prevalence of hypertension in the first, second, third, and fourth quartiles of Ln(SII).

SII: systemic immunity-inflammation index.

Tertile 1: 0-5.840;

Tertile 2: 5.841-6.181;

Tertile 3: 6.182-6.527;

Tertile 4: >6.527.

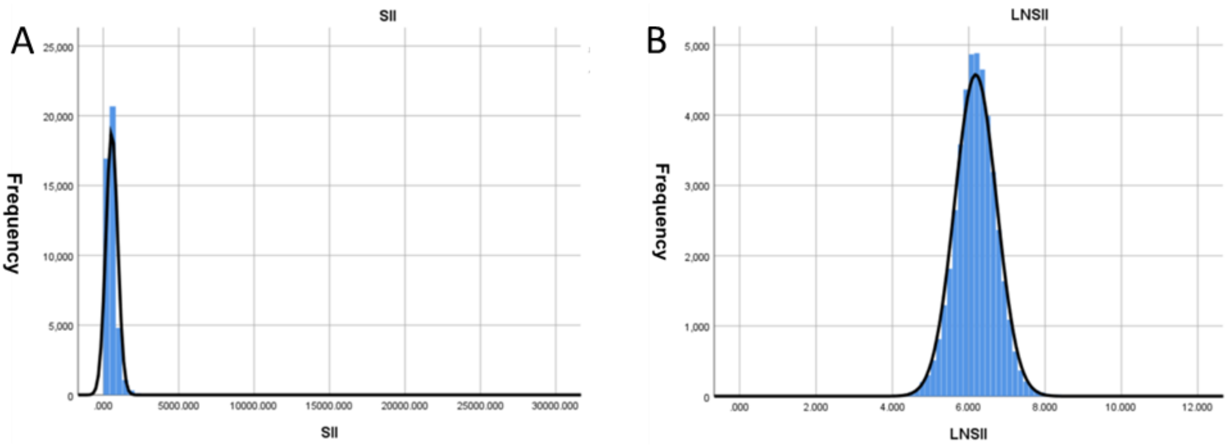

Supplementary Figure 2. The distribution of SII among all participants (A) and the distribution after Ln-conversion (B)

SII: systemic immunity-inflammation index.
