# Supplementary material for: Association between systemic immunity-inflammation index and hypertension in US adults from NHANES 1999–2018
Source: Sci Rep. 2024 Mar 7;14:5677. doi: 10.1038/s41598-024-56387-6 (PMC10920861; doi:10.1038/s41598-024-56387-6)
Supplement: Supplementary file 3 — Supplementary Table 1. [file 41598_2024_56387_MOESM3_ESM.pdf]

Supplementary Table 1 Unweighted Logistic Regression Analysis of Ln (SII) and Hypertension.

	Model 1		Model 2		Model 3	
	OR(95%CI)	P	OR(95%CI)	P	OR(95%CI)	P
Continuous	1.11(1.07,1.15)	<0.001	1.08(1.04,1.12)	<0.001	1.09(1.04,1.13)	<0.001
Categories						
Tertile 1	Ref.		Ref.		Ref.	
Tertile 2	0.96(0.90,1.01)	0.111	0.96(0.90,1.02)	0.878	1.03(0.96,1.10)	0.525
Tertile 3	1.03(0.97,1.09)	0.333	1.04(0.98,1.10)	0.029	1.09(1.02,1.17)	0.101
Tertile 4	1.17(1.11,1.23)	<0.001	1.11(1.05,1.18)	<0.001	1.12(1.04,1.20)	0.027
P for trend		<0.001		<0.001		0.198

Log(SII), Tertile 1: 0-5.840; Tertile 2:5.841-6.181; Tertile 3:6.182-6.527; Tertile 4:>6.527.

SII: Systemic immune inflammation index;
